# C-reactive protein cut-offs used for acute respiratory infections in Danish general practice

**DOI:** 10.3399/bjgpopen20X101136

**Published:** 2021-01-27

**Authors:** Jesper Lykkegaard, Jonas Kanstrup Olsen, Rikke Vognbjerg Sydenham, Malene Plejdrup Hansen

**Affiliations:** 1 Research Unit for General Practice, Institute of Public Health, University of Southern Denmark, Odense, Denmark; 2 Audit Project Odense, Research Unit for General Practice, Institute of Public Health, University of Southern Denmark, Odense, Denmark; 3 Center for General Practice, Aalborg University, Aalborg, Denmark

**Keywords:** Respiratory tract infections, C-reactive protein, Antibiotics, General practice, Diagnostics

## Abstract

**Background:**

GPs can use the C-reactive protein (CRP) point-of-care test (POCT) to assist when deciding whether to prescribe antibiotics for patients with acute respiratory tract infections (RTIs).

**Aim:**

To estimate the CRP cut-off levels that Danish GPs use to guide antibiotic prescribing for patients presenting with different signs and symptoms of RTIs.

**Design & setting:**

A cross-sectional study conducted in general practice in Denmark.

**Method:**

During the winters of 2017 and 2018, 143 GPs and their staff registered consecutive patients with symptoms of an RTI according to the Audit Project Odense (APO) method. CRP cut-offs were estimated as the lowest level at which half of the patients were prescribed an antibiotic.

**Results:**

In total, 7813 patients were diagnosed with an RTI, of whom 4617 (59%) had a CRP test performed. At least 25% of the patients were prescribed an antibiotic when the CRP level was >20 mg/L, at least 50% when CRP was >40 mg/L, and at least 75% when CRP was >50 mg/L. Lower thresholds were identified for patients aged ≥65 years and those presenting with a fever, poor general appearance, dyspnoea, abnormal lung auscultation, or ear/facial pain, and if the duration of symptoms was either short (≤1 day) or long (>14 days).

**Conclusion:**

More than half of patients presenting to Danish general practice with symptoms of an RTI have a CRP test performed. At CRP levels >40 mg/L, the majority of patients have an antibiotic prescribed.

## How this fits in

For more than 20 years, GPs in Denmark have been paid a fee for performing a CRP test. No specific CRP cut-off level exists when using CRP to assist decisions on whether to prescribe antibiotics for patients with an RTI, and GPs are encouraged to always evaluate the result in combination with careful history taking and a physical examination.

This study shows that more than half of patients attending Danish general practice with symptoms of an acute RTI have a CRP test performed, and 40 mg/L is the CRP-level above which the majority of patients have an antibiotic prescribed. That level varies substantially with the patients’ age, signs, and symptoms indicating that CRP results are evaluated in the context of other information.

## Introduction

CRP is a marker of inflammation that can be measured quickly in general practice as a POCT.^1,2^ The level of CRP ranges from around 2 mg/L in healthy patients to as high as 500 mg/L in patients with a severe inflammatory response.^3^ The role of CRP POCT in distinguishing bacterial from viral infections in general practice is much debated.^4–6^ An elevated CRP level (>20 mg/L) is associated with having an infiltrate on a chest radiograph.^7–9^ Furthermore, elevated CRP levels have been associated with the diagnosis of maxillary sinusitis^10^ and with benefits from antibiotic treatment in patients with exacerbation of chronic obstructive pulmonary disease.^11^ However, a systematic review concluded that CRP POCT was not sufficiently sensitive to rule out, nor sufficiently specific to rule in, a bacterial lower RPI.^12^

GPs in many countries use CRP POCT to assist when deciding whether to prescribe antibiotics for patients with acute RPIs. Use of CRP POCT in general practice can reduce antibiotic prescription and thereby help slow down the worrying rise in antibiotic resistant bacteria.^13–17^ The use of CRP POCT is expected to rapidly increase globally.^18^

Most randomised trials on the effects of introducing CRP POCT in general practice have used the recommendations not to prescribe antibiotics if CRP is <20 mg/L, and to prescribe antibiotics if CRP ≥100 mg/L.^15,17,19–21^ In addition, some of the trials recommended withholding antibiotics in most cases if CRP <50 mg/L.^17,19–21^ Guidelines in most countries, including the UK, the Netherlands, and the Scandinavian countries, tend to follow these recommendations.^22–24^

Around 20% of CRP test results range from 20 to 99 mg/L, where they offer limited assistance to the GP.^15,19^ There is little knowledge on how GPs in their daily practice interpret these intermediate levels of CRP and overcome the dilemma about when to prescribe antibiotics. For more than 20 years, the GPs in Denmark have been paid a fee for performing a CRP POCT, and virtually all Danish general practices use CRP on a daily basis. This study aimed to estimate which CRP cut-off levels Danish GPs use to guide antibiotic prescribing for patients presenting with different signs and symptoms of acute RPIs.

## Method

### Design

All GPs and practice staff in the northern, southern, and central regions of Denmark were invited to register all consecutive consultations in which the patient presented with symptoms of an acute RPI. Healthcare professionals registered patients during four weeks in the winters of 2017 or 2018.

### Setting

Denmark has 5.8 million citizens, 54% living in the three study regions. In Denmark, family medicine is a specialty in line with other medical specialties. To become a GP, it requires authorisation from the Danish Board of Health based on 5 years of specialist training and courses. GPs manage the vast majority of patients with acute RTIs and act as gatekeeper to secondary care treatment. GPs are self-employed, working on a contract with the public funder. Nearly all services to the patients are tax paid. The GPs receive a capitation fee and fees for services, including a fee for measuring CRP (€9), paying for the test equipment themselves. All antibiotics are on prescription only. The use of antibiotics in Denmark is below the EU average.^25^

### Data

Data were collected by means of a simple registration chart provided by APO (Supplementary Figure 1). For each patient, the healthcare professional registered symptoms, duration, if a deterioration occurred, findings, the level of CRP (if measured), and if antibiotics were prescribed.

### Analysis

Descriptive statistics and age-adjusted logistic regression were used to analyse associations between patient characteristics and whether a CRP POCT was performed.

**Figure 1.**
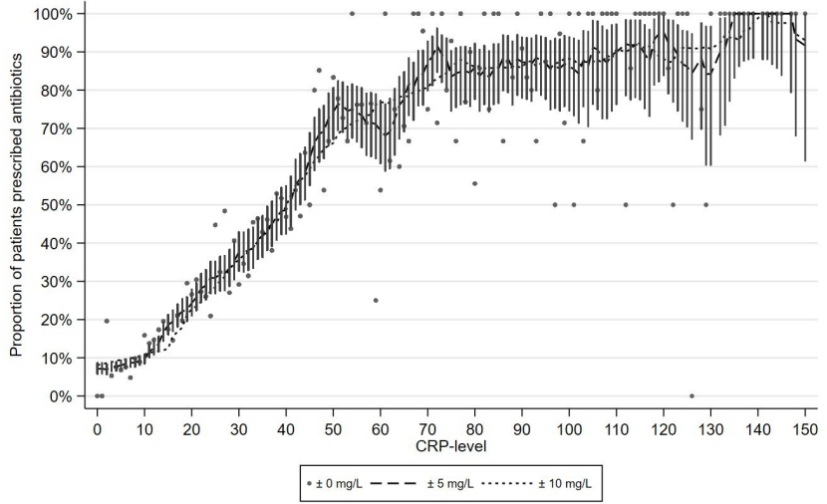
C-reactive protein (CRP) levels and the proportion of patients prescribed an antibiotic. Legends indicate the width of the interval around the index CRP level used to calculate the proportion of patients who were prescribed antibiotics, respectively 0, 5, and 10mg/L above and below. Bars indicate 95% confidence intervals. CRP levels 150–300mg/L not shown.

For each level of CRP, the proportion of patients who were prescribed an antibiotic was calculated based on the patients who had a CRP level in the range of 5 mg/L below the level to 5 mg/L above. Based on binomial distribution, 95% confidence intervals (CI) were calculated for each level of CRP. The CRP cut-off level for prescribing antibiotics was defined as the lowest level at which at least half of the patients were prescribed an antibiotic. The authors performed sensitivity analyses using ranges, 0 mg/L and 10 mg/L below and above the index CRP level, respectively (Figure 1). All analyses were performed in STATA Release 15 (STATACorp, College Station, TX, USA).

## Results

A total of 143 GPs participated in the study; 20% of all GPs in the northern, 6% in the southern, and 5% in the central region of Denmark (Table 1). The GPs and practice staff registered a total of 8232 patients diagnosed with an acute RTI. After removing patients with missing information on CRP or antibiotics, the study population comprised 7832 patients, of whom 4635 (59%) had a CRP POCT performed.

**Table 1.** Characteristics of the 143 participating GPs compared to all GPs in each regions.

	**Northern region, *n* (%**)	**Southern region, *n* (%**)	**Central region, *n* (%**)
	All GPs^a^	Participants	All GPs^a^	Participants	All GPs^a^	Participants
Total	303	59 (20)	785	46 (5.9)	811	38 (4.7)
Mean age, years	54	49	59	49	52	51
Female GPs	134 (44)	35 (59)	385 (49)	30 (65)	427 (53)	24 (63)

^a^Numbers from the Organisation of General Practitioners^32^

CRP POCT was more often performed on patients presenting with dyspnoea (78%, odds ratio (OR) 1.97 [95% CI = 1.63 to 2.36]), cough (66%, OR 2.85 [95% CI = 2.54 to 3.20]), or an abnormal lung auscultation (72%, OR 1.67 [95% CI = 1.42 to 1.97]) than on patients with a sore throat (56%, OR 0.78 [95% CI = 0.69 to 0.88]) or ear/facial pain (45%, OR 0.54 [95% CI = 0.47 to 0.62]). Furthermore, having a CRP POCT performed was associated with increasing age and patients presenting with a fever, deterioration of symptoms, or being assessed with a poor general appearance (Table 2).

**Table 2.** C-reactive protein (CRP) tests for patients with acute respiratory tract infections in Danish general practice

Patient characteristics	Total	CRP POCT performed	Odds of CRP POCT
*n* (%)	*n* (%)	OR_age-adjusted_ (95% CI)
Total	7832 (100)	4635 (59)	
**Age groups, years**			
0–14	2705 (35)	772 (29)	1 (reference)
15–64	3793 (49)	2773 (73)	6.81 (6.10 to 7.60)
>64	1305 (17)	1071 (82)	11.46 (9.72 to 13.51)
**Symptoms and findings**			
Fever	2971 (38)	1811 (61)	1.81 (1.62 to 2.02)
Cough	5611 (72)	3714 (66)	2.85 (2.54 to 3.20)
Sore throat	1866 (24)	1053 (56)	0.78 (0.69 to 0.88)
Ear/face pain	1317 (17)	592 (45)	0.54 (0.47 to 0.62)
Dyspnoea	881 (11)	691 (78)	1.97 (1.63 to 2.36)
Purulent rhinorrhoea	1324 (17)	714 (54)	0.83 (0.72 to 0.94)
Deterioration of symptoms	861 (11)	569 (66)	1.34 (1.13 to 1.58)
Poor general appearance	1546 (21)	1131 (73)	2.15 (1.87 to 2.47)
Abnormal lung auscultation	1063 (14)	765 (72)	1.67 (1.42 to 1.97)
**Duration of symptoms, days**			
≤1	504 (7)	193 (38)	1 (reference)
2–4	2958 (40)	1551 (52)	1.35 (1.09 to 1.68)
5–14	3233 (44)	2134 (66)	1.85 (1.49 to 2.30)
>14	679 (9)	470 (69)	1.60 (1.22 to 2.09)

CI = confidence interval. CRP = C-reactive protein. OR = odds ratio. POCT = point-of-care test

The quarter of GP clinics with the highest use of CRP POCT each tested >71% of their patients and prescribed antibiotics to 27%, while the quarter of clinics with the lowest use of CRP POCT tested <52% of their patients and prescribed antibiotics to 29%.

**Figure 2.**
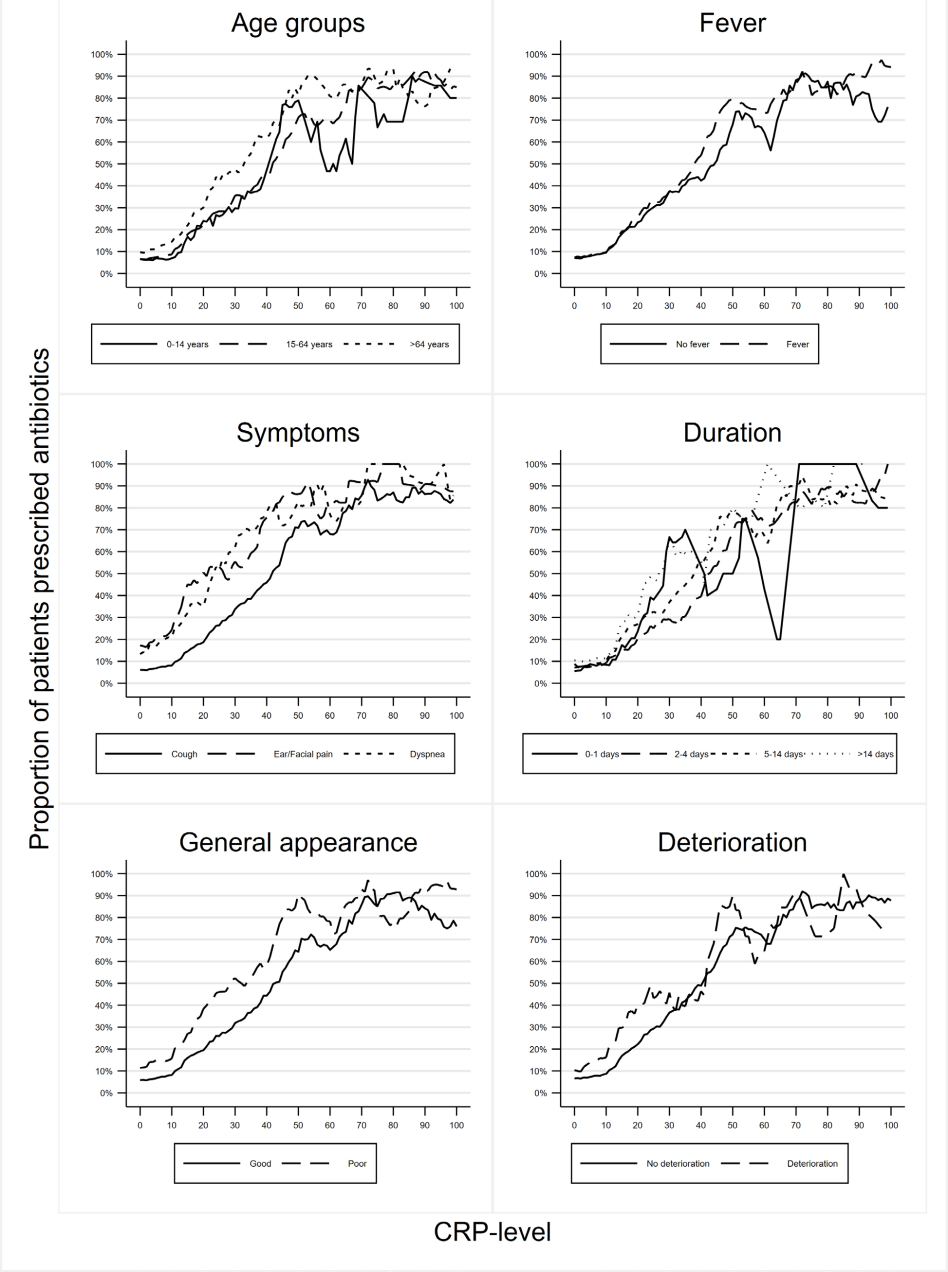
Proportions of patients who are prescribed an antibiotic according to C-reactive protein (CRP) levels in the range from 5 mg/L below to 5 mg/L above the index. CRP levels 100–300 mg/L not shown.

Among the patients who had a CRP POCT, at least 25% were prescribed an antibiotic when the CRP level was >20 mg/L (95% CI = 18 to 22 mg/L), at least 50% when CRP was >40 mg/L (95% CI = 37 to 42 mg/L), and at least 75% when CRP was >50 mg/L (95% CI = 47 to 98 mg/L). These CRP levels varied only little when changing the width of the CPR intervals used to estimate the antibiotics prescription proportions (Figure 1). Lower thresholds were identified for patients aged ≥65 years and those presenting with a fever, poor general appearance, dyspnoea, abnormal lung auscultation, or ear/facial pain, and if the duration of symptoms was either short (≤1 day) or long (>14 days) (Table 3 and Figure 2).

**Table 3.** C-reactive protein (CRP) cut-offs^a^ used by 143 Danish GPs for patients with acute respiratory tract infections

Patient characteristics	25% cut-offCRP, mg/L (CI)	50% cut-offCRP, mg/L (CI)	75% cut-offCRP, mg/L (CI)
All the patients (*n* = 4635)	20 (18 to 22)	40 (37 to 42)	50 (47 to 98)
**Age group, years**			
0–14	22 (18 to 67)	43 (32 to 98)	45 (43 to 98)
15–64	22 (20 to 28)	42 (38 to 45)	64 (49 to 99)
>64	17 (14 to 20)	33 (24 to 41)	46 (41 to 98)
**Fever**			
Yes	20 (17 to 23)	38 (34 to 41)	47 (45 to 84)
No	22 (17 to 25)	45 (36 to 97)	66 (50 to 99)
**Symptoms**			
Cough	24 (22 to 27)	42 (38 to 44)	64 (49 to 99)
Sore throat	29 (19 to 37)	45 (38 to 71)	74 (49 to 99)
Ear/face pain	11 (5 to 12)	20 (14 to 99)	40 (37 to 99)
Dyspnoea	11 (8 to 20)	24 (21 to 99)	38 (30 to 99)
Purulent rhinorrhoea	14 (13 to 32)	38 (25 to 99)	55 (40 to 99)
**Duration of symptoms, days**			
≤1	21 (14 to 89)	29 (22 to 99)	53 (29 to 99)
2–4	24 (20 to 36)	43 (41 to 48)	54 (49 to 98)
5–14	17 (15 to 22)	38 (33 to 61)	46 (45 to 99)
>14	15 (14 to 93)	27 (21 to 93)	50 (29 to 93)
**Deterioration of symptoms**			
Yes	14 (11 to 39)	42 (20 to 99)	46 (42 to 99)
No	22 (20 to 24)	41 (37 to 44)	51 (49 to 98)
**Poor general appearance**			
Yes	15 (11 to 17)	29 (22 to 41)	44 (42 to 85)
No	24 (21 to 29)	43 (39 to 46)	66 (51 to 99)
**Abnormal lung auscultation**			
Yes	3 (1 to 8)	22 (13 to 27)	32 (28 to 99)
No	24 (21 to 33)	45 (42 to 62)	68 (50 to 99)

^a^Estimated as the lowest level of CRP at which the given percentage of patients were prescribed an antibiotic. Values in brackets indicate the 95% CI for the CRP levels ±5 mg/L, based on binomial distribution. CI = confidence interval. CRP = C-reactive protein.

## Discussion

### Summary

A total of 59% of patients attending Danish general practice with symptoms of an acute RTI had a CRP test performed. No use of any strict CRP cut-off level for antibiotic prescribing was identified. However, at least half of the patients were prescribed an antibiotic when the level was >40 mg/L. The GPs’ interpretation of the CRP levels depended on the patient’s age, symptoms, and signs. Lower CRP thresholds were identified for older patients and those presenting with a fever, poor general appearance, dyspnoea, abnormal lung auscultation, or ear/facial pain, and if the duration of symptoms was short (≤1 day) or long (>14 days).

### Strengths and limitations

Fewer than one in 10 eligible GPs participated in the study. It is likely that these GPs are more interested in the management of patients with acute RTIs and thus differ in the use and interpretation of CRP tests compared to the non-participants. Furthermore, Danish GPs have a lower antibiotic prescription rate and a longer tradition for the use of CRP tests compared to GPs in many other countries.^25^ Consequently, the identified cut-off CRP levels are likely to be different if studied in another country.

As demonstrated the CRP cut-off estimates depend heavily on the case-mix, for example, patient age, symptoms, and signs. The participating healthcare professionals were asked to consecutively include all patients with symptoms of an acute RTI making selection in the GP surgery less likely. However, local incidence rates of the different acute RTIs at the time of the study and the patients’ doctor-seeking behaviour strongly influence the type of patients included. If fewer mild cases presented in the GP surgery, the proportion of severe cases would increase, and probably also the proportion of patients having a CRP tests performed and being prescribed an antibiotic. More important to the study aim, inclusion of more patients with any of the characteristics associated with a low cut-off threshold would lower both the overall threshold estimate and the thresholds found in patients with all other characteristics.

The GPs reported 6% of the patients to have explicitly asked for a POCT, but the registration chart did not distinguish between asking for a CRP or a streptococcus A rapid test.

Lastly, the cross-sectional design impairs all interpretations of causality. Decisions to record a symptom or finding may be influenced by the measured CRP level or a prior decision on whether to prescribe an antibiotic. Still, the risk of information bias was considered low since Danish GPs and practice staff are familiar with the APO template and made the recordings for their own voluntary quality improvement purposes.

### Comparison with existing literature

Many guidelines exist on when to prescribe antibiotics according to the CRP level for patients with acute RTIs. Most agree that if the CRP level is <20 mg/L antibiotics should not be prescribed. However, "the upper value" in support of antibiotic treatment differ between guidelines. Importantly, most studies on the use of CRP tests in patients with acute RTIs have been performed in patients with acute cough, and solid evidence is lacking for its use in patients with other infections.^26^

Qualitative studies have identified that GPs request guidance on the interpretation of CRP values.^27,28^ The Danish College of General Practitioners’ guideline for managing patients with acute RTIs state that if the CRP value is >50 mg/L, antibiotics should be considered.^24^ The present study indicates that Danish GPs align with that recommendation.

Both national and international guidelines highlight that the result of a CRP test always must be evaluated together with the patient history and examination. However, in Scandinavia CRP POCT is increasingly used^29,30^ and sometimes performed even before the patient has been assessed clinically (Bisgaard *et al*, unpublished data, 2021). This may explain why compared to being assessed with an abnormal lung auscultation, the symptom “cough” was closer associated with having a CRP POCT performed. In the worst case, overuse of the CRP test can result in diagnostic uncertainty and consequently lead to an overuse of antibiotics.^31^

A recent editorial by Cals and Ebell discussed how the CRP test might be incorporated into clinical decision rules by combining the test result with symptoms and findings.^26^ Correspondingly, the authors found that older patients and those with a fever, dyspnoea, abnormal lung auscultation, ear/facial pain, and those with a poor general appearance were prescribed antibiotics at lower CRP levels than other patients.

### Implications for research and practice

Antibiotic use is the main driver of antimicrobial resistance. Solid evidence exists that use of CRP POCT can reduce antibiotic prescribing for acute RTIs. This study found that more than half of the patients had a CRP test performed. This was even among those presenting with a sore throat, where the streptococcus A rapid test is recommended rather than the CRP POCT.^23^ Many of the patients with a sore throat had other symptoms, as well, and having the sore throat reduced their chance of having a CRP POCT performed. Nevertheless, these findings suggest a substantial overuse of CRP POCT in Denmark.

Perhaps now the field has reached a time where future studies need to focus less on the CRP tests’ ability to reduce antibiotic prescribing, and more on when the test is indicated and how the test results are interpreted. No prior observation studies have investigated how CRP cut-off levels are applied in daily clinical practice. The presented CRP levels are useful as a reference for GPs in Denmark, and other countries, while waiting for evidence-based algorithms integrating CRP levels with signs and symptoms in guiding antibiotic prescriptions.

## References

[1] Volanakis JE (2001). Human C-reactive protein: expression, structure, and function. Mol Immunol.

[2] Seamark DA, Backhouse SN, Powell R (2003). Field-testing and validation in a primary care setting of a point-of-care test for C-reactive protein. Ann Clin Biochem.

[3] Falk G, Fahey T (2009). C-Reactive protein and community-acquired pneumonia in ambulatory care: systematic review of diagnostic accuracy studies. Fam Pract.

[4] Hopstaken RM, Stobberingh EE, Knottnerus JA (2005). Clinical items not helpful in differentiating viral from bacterial lower respiratory tract infections in general practice. J Clin Epidemiol.

[5] Almirall J, Bolíbar I, Toran P (2004). Contribution of C-reactive protein to the diagnosis and assessment of severity of community-acquired pneumonia. Chest.

[6] Macfarlane J, Holmes W, Gard P (2001). Prospective study of the incidence, aetiology and outcome of adult lower respiratory tract illness in the community. Thorax.

[7] Hopstaken RM, Muris JW, Knottnerus JA (2003). Contributions of symptoms, signs, erythrocyte sedimentation rate, and C-reactive protein to a diagnosis of pneumonia in acute lower respiratory tract infection. Br J Gen Pract.

[8] Holm A, Pedersen SS, Nexoe J (2007). Procalcitonin versus C-reactive protein for predicting pneumonia in adults with lower respiratory tract infection in primary care. Br J Gen Pract.

[9] Htun TP, Sun Y, Chua HL, Pang J (2019). Clinical features for diagnosis of pneumonia among adults in primary care setting: a systematic and meta-review. Sci Rep.

[10] Hansen JG, Schmidt H, Rosborg J, Lund E (1995). Predicting acute maxillary sinusitis in a general practice population. BMJ.

[11] Llor C, Moragas A, Hernández S (2012). Efficacy of antibiotic therapy for acute exacerbations of mild to moderate chronic obstructive pulmonary disease. Am J Respir Crit Care Med.

[12] van der Meer V, Neven AK, van den Broek PJ, Assendelft WJJ (2005). Diagnostic value of C reactive protein in infections of the lower respiratory tract: systematic review. BMJ.

[13] Tonkin-Crine SK, Tan PS, van Hecke O (2017). Clinician-targeted interventions to influence antibiotic prescribing behaviour for acute respiratory infections in primary care: an overview of systematic reviews. Cochrane Database Syst Rev.

[14] Aabenhus R, Jensen J-US, Jørgensen KJ (2014). Biomarkers as point-of-care tests to guide prescription of antibiotics in patients with acute respiratory infections in primary care. Cochrane Database Syst Rev.

[15] Cals JWL, Schot MJC, de Jong SAM (2010). Point-of-care C-reactive protein testing and antibiotic prescribing for respiratory tract infections: a randomized controlled trial. Ann Fam Med.

[16] Verbakel JY, Lee JJ, Goyder C (2019). Impact of point-of-care C reactive protein in ambulatory care: a systematic review and meta-analysis. BMJ Open.

[17] Do NTT, Ta NTD, Tran NTH (2016). Point-of-care C-reactive protein testing to reduce inappropriate use of antibiotics for non-severe acute respiratory infections in Vietnamese primary health care: a randomised controlled trial. Lancet Glob Health.

[18] Sohn AJ, Hickner JM, Alem F (2016). Use of point-of-care tests (POCTs) by US primary care physicians. J Am Board Fam Med.

[19] Cals JWL, Butler CC, Hopstaken RM (2009). Effect of point of care testing for C reactive protein and training in communication skills on antibiotic use in lower respiratory tract infections: cluster randomised trial. BMJ.

[20] Andreeva E, Melbye H (2014). Usefulness of C-reactive protein testing in acute cough/respiratory tract infection: an open cluster-randomized clinical trial with C-reactive protein testing in the intervention group. BMC Fam Pract.

[21] Little P, Stuart B, Francis N (2013). Effects of internet-based training on antibiotic prescribing rates for acute respiratory-tract infections: a multinational, cluster, randomised, factorial, controlled trial. Lancet.

[22] National Institute for Health and Care Excellence (2014). Pneumonia: diagnosis and management of community- and hospital-acquired pneumonia in adults. https://www.nice.org.uk/guidance/CG191/documents/pneumonia-guideline-consultation-nice-guideline2.

[23] Verlee L, Verheij TJM, Hopstaken RM (2012). Summary of NHG practice guideline 'acute cough'. Ned Tijdschr Geneeskd.

[24] Danish College of General Practitioners (2014). Airway infections — diagnosis and treatment] *Luftvejsinfektioner – diagnose og behandling* (in Danish). https://vejledninger.dsam.dk/luftvejsinfektioner/.

[25] European Centre for Disease Prevention and Control (2019). Antimicrobial consumption in the EU/EEA, annual epidemiological report for 2018. https://www.ecdc.europa.eu/en/publications-data/surveillance-antimicrobial-consumption-europe-2018.

[26] Cals JWL, Ebell MH (2018). C-Reactive protein: guiding antibiotic prescribing decisions at the point of care. Br J Gen Pract.

[27] Hardy V, Thompson M, Keppel GA (2017). Qualitative study of primary care clinicians' views on point-of-care testing for C-reactive protein for acute respiratory tract infections in family medicine. BMJ Open.

[28] Cals JWL, Chappin FHF, Hopstaken RM (2010). C-Reactive protein point-of-care testing for lower respiratory tract infections: a qualitative evaluation of experiences by GPs. Fam Pract.

[29] Moberg AB, Cronberg O, Falk M, Hedin K (2020). Change in the use of diagnostic tests in the management of lower respiratory tract infections: a register-based study in primary care. BJGP Open.

[30] Haldrup S, Thomsen RW, Bro F (2017). Microbiological point of care testing before antibiotic prescribing in primary care: considerable variations between practices. BMC Fam Pract.

[31] Lemiengre MB, Verbakel JY, Colman R (2018). Reducing inappropriate antibiotic prescribing for children in primary care: a cluster randomised controlled trial of two interventions. Br J Gen Pract.

[32] PLO (2019). [Organisation of General Practitioners in Denmark fact sheet] *Praktiserende lægers organisation fakta ark* (in Danish). https://www.laeger.dk/sites/default/files/plo_faktaark_2018.pdf.

